# Supplementary material for: The Mechanism of TT2-Type MYB Transcription Factor JrMYB1L in Anthocyanin Biosynthesis in ‘Jinghong 1’ Walnuts
Source: Plants (Basel). 2025 Dec 6;14(24):3727. doi: 10.3390/plants14243727 (PMC12737103; doi:10.3390/plants14243727)
Supplement: Supplementary file 1 [file plants-14-03727-s001.zip › Supplementary materials/Supplementary figures.pdf]

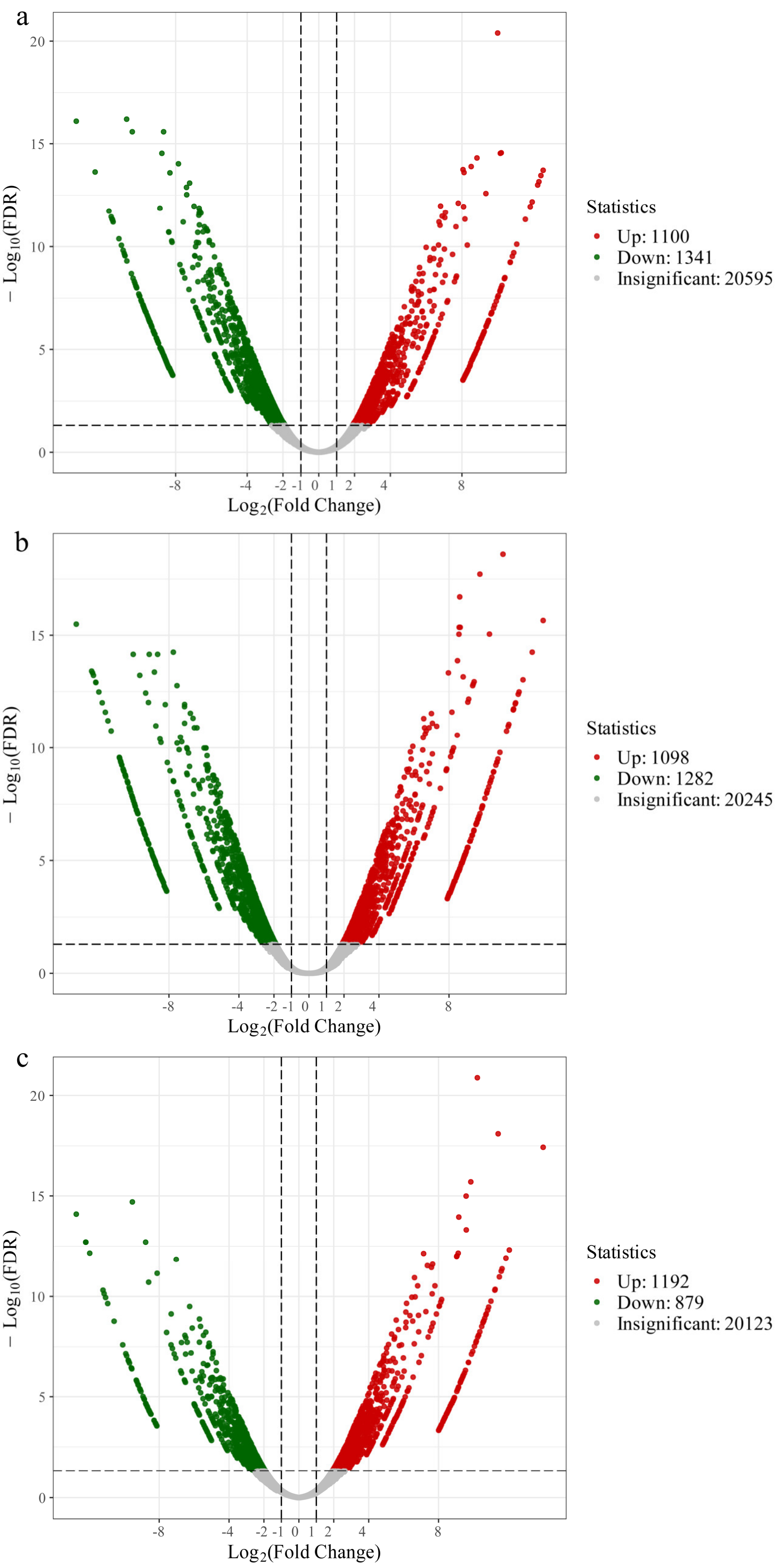

Figure S1. The distribution of differentially expressed genes in the testa of 'D2-1' and 'Jinghong 1'. (a–c) represent differentially expressed genes in the testa of 'D2-1' and 'Jinghong 1' in the green fruit, testa color transition, and ripening stages, respectively.

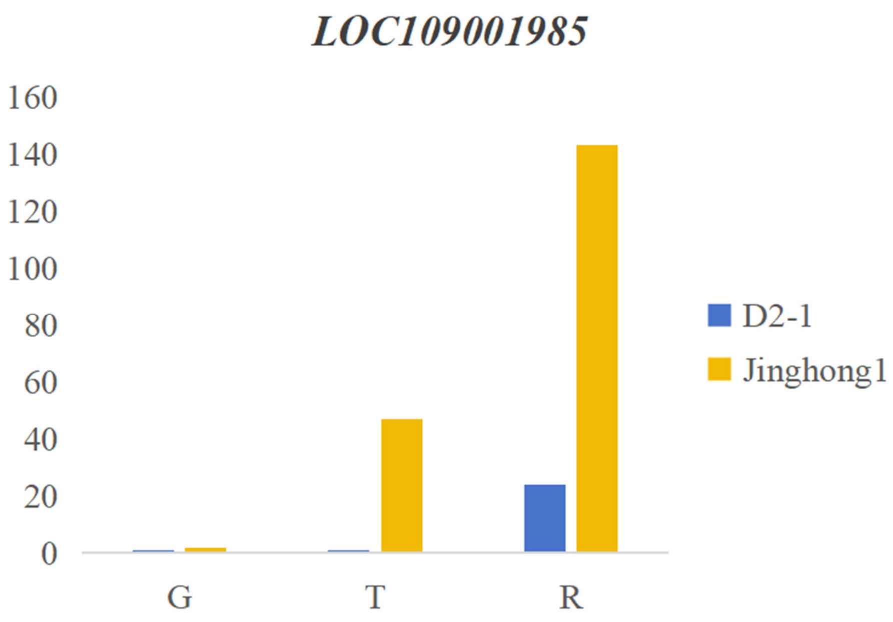

Figure S2. Expression characteristics of *LOC109001985* in ‘D2-1’ and ‘Jinghong 1’ testa. Note: G, T, and R represent green fruit, testa color transition, and ripening stages, respectively.

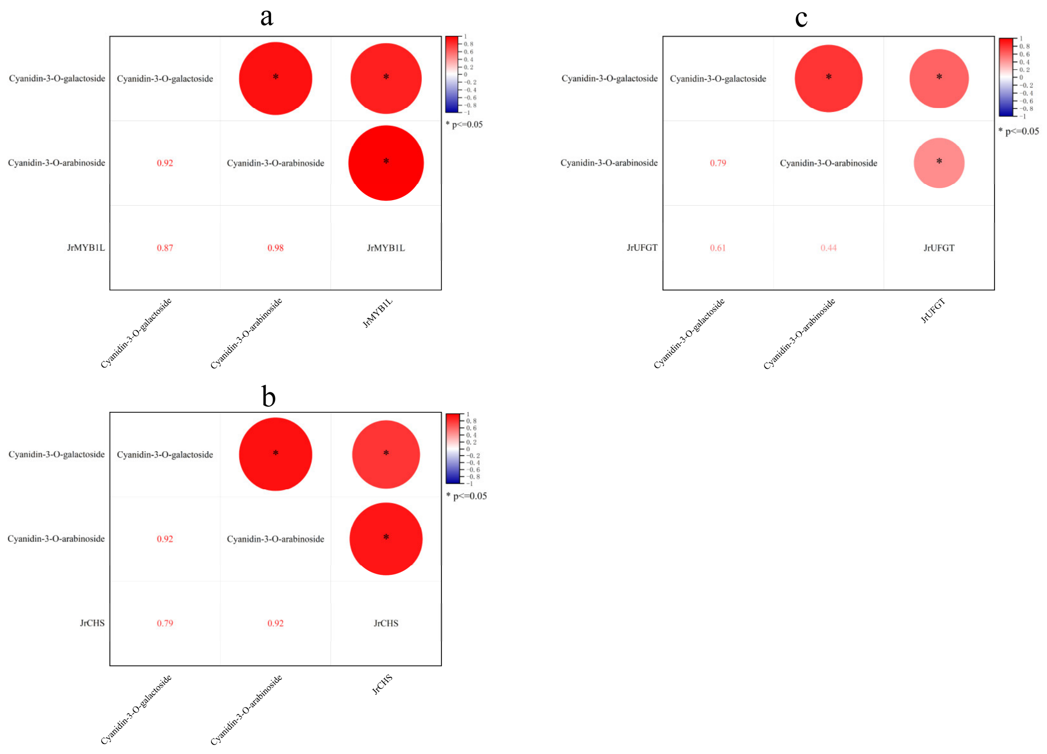

Figure S3. The correlation among *JrMYB1L* (a), *JrCHS* (b), and *JrUFGT* (c) expression and the content of cyanidin-3-O-galactoside and cyanidin-3-O-arabinoside.

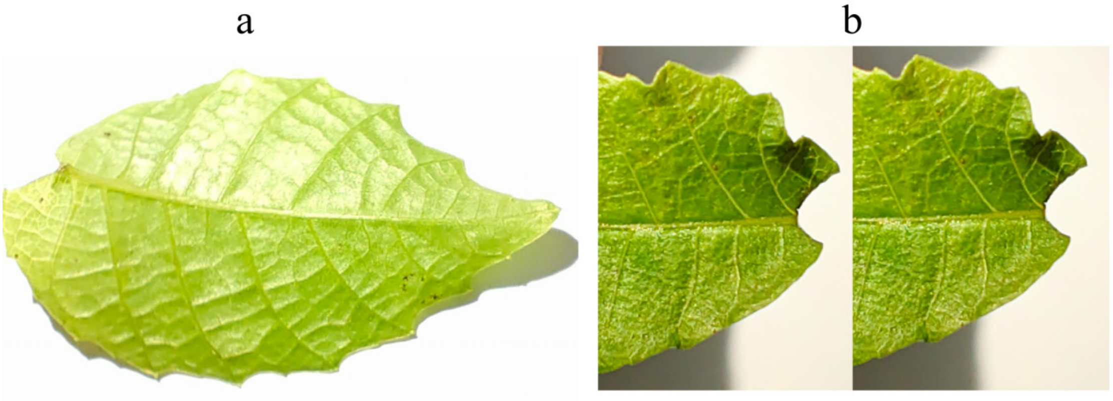

Figure S4. Phenotypic traits of walnut leaves the control group (a) and the *JrMYB1L*-overexpressed group (b).

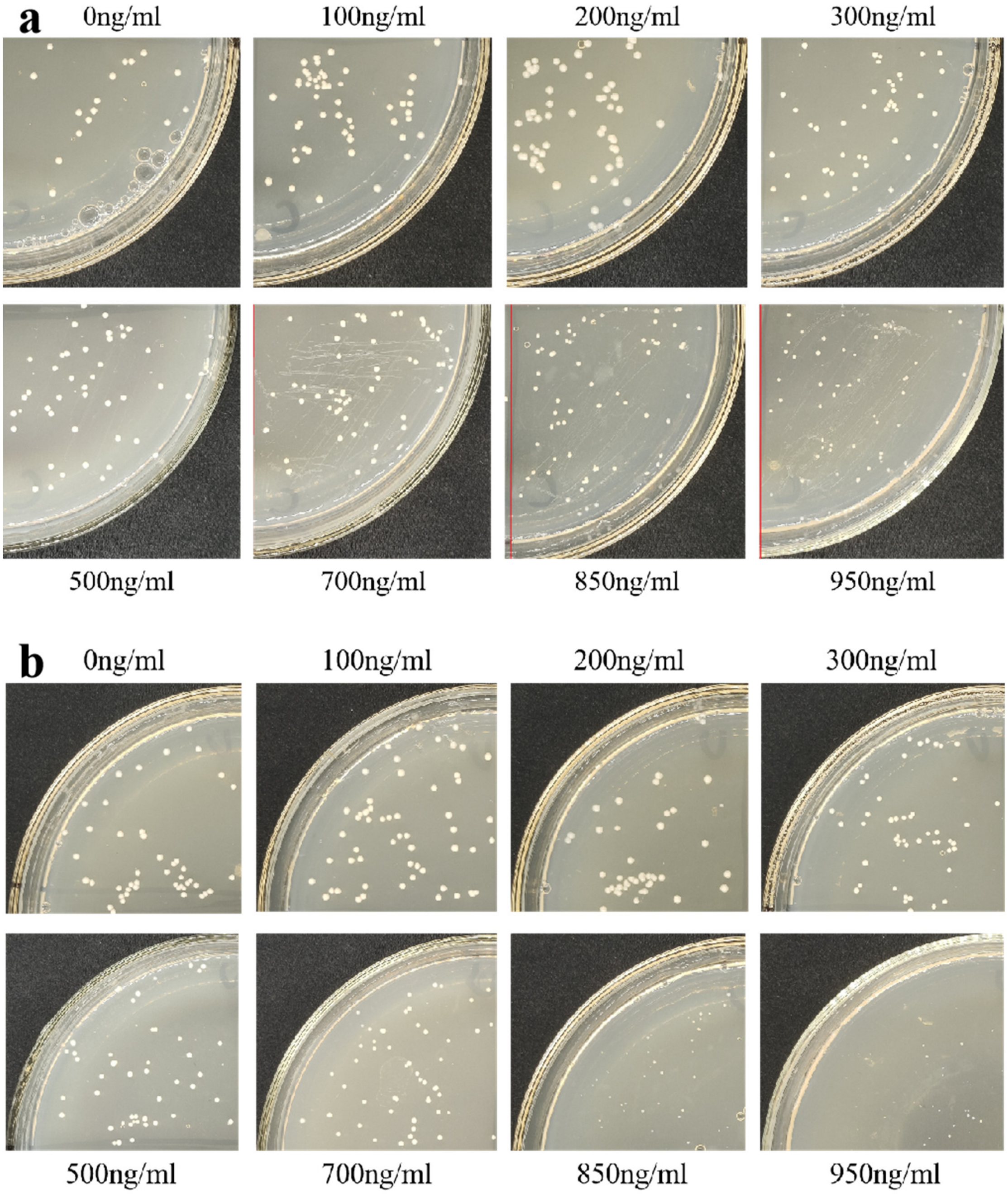

Figure S5. Suppression of auto-activation activity by *JrCHSpro1427-1509* (a) and *JrUFGTpro1200-1400* (b).

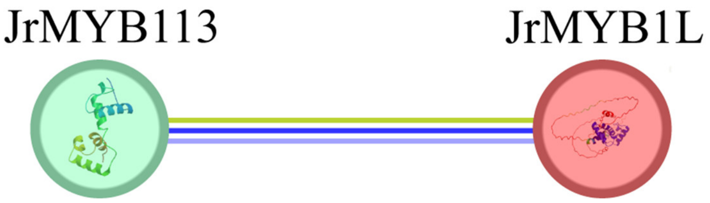

Figure S6. Computational prediction of JrMYB113-JrMYB1L protein interaction.
